# Intranasal corticosteroid users in The Netherlands: A drug utilization study

**DOI:** 10.1016/j.jacig.2024.100303

**Published:** 2024-07-20

**Authors:** Corine Rollema, Eric N. Van Roon, Nynke C.C.M. Schuiling-Veninga, Jens H.J. Bos, Tjalling W. De Vries

**Affiliations:** a Department of Clinical Pharmacy and Pharmacology, Medical Centre Leeuwarden, Leeuwarden, The Netherlands; b Groningen Research Institute of Pharmacy, Department Pharmacotherapy, Epidemiology, and Economy, University of Groningen, Groningen, The Netherlands; c Department of Paediatrics, Medical Centre Leeuwarden, Leeuwarden, The Netherlands

**Keywords:** Intranasal corticosteroids, administration technique, patient-tailored administration instructions, prescription data, characteristics, comedication

## Abstract

**Background:**

To improve (patient-tailored) instructions for intranasal corticosteroid (INC) administration, we need to gain insight into specific characteristics of INC users and comedication use.

**Objective:**

We examined INC prescriptions obtained from the Dutch InterAction Database to gain insight into the prevalence and incidence rates, INC use in previous years, and comedication.

**Methods:**

We retrospectively examined INC prescriptions written between January 1, 2015, and December 31, 2019. Prevalence and incidence rates were stratified by age and sex. The use of INCs in previous years and comedication were analyzed.

**Results:**

In 2019, a total of 172,563 INC prescriptions were written and dispensed to 75,048 individuals. Also in 2019, the prevalence and incidence of INC users were 68.9 and 25.6 per 1000 individuals, respectively. INCs were used by all age groups. More than half of INC users in 2019 did not receive a prescription in 2018, almost a quarter received a prescription in 5 consecutive years, 28% used an INC in combination with an inhaler, 29% used an INC together with a systemic antihistamine, 9% used an INC along with ocular medication, and 1% used an INC with an intranasal antihistamine. Several corticosteroid-containing drugs were being used in combination with INCs by 2% to 16% of those studied.

**Conclusion:**

This study gives insights into opportunities for patient-tailored instructions. INCs are used by various age groups and by new or intermittent users as well as by continuous users. On the bases of these results, patient-tailored instructions can be developed and subsequently studied to determine whether the instructions affect treatment adherence and efficacy. The insights gained about comedication provide opportunities for improved evaluation of the INC administration technique. Taken together, these suggestions might lead to a more patient-tailored approach, which might in turn lead to improved treatment with INCs.

Intranasal corticosteroids (INCs) are prescribed for allergic rhinitis (AR) and for rhinosinusitis complaints accompanied by nasal polyps, among other conditions.^1^, ^2^, ^3^ Uncontrolled AR and sinusitis symptoms may negatively influence patient’s quality of life and affect health care costs.^4^^,^^5^ Adequate treatment is necessary, and INCs have proved to be effective: INCs reduce nasal and ocular symptoms and improve quality of life.^3^^,^^5^^,^^6^ The technique for administration of INCs may influence the deposition pattern of INC particles on the nasal mucosa and thereby affect pharmacologic efficacy, safety, compliance, and patient satisfaction.^7^, ^8^, ^9^ Improvement of INC administration would be valuable for better treatment of AR and rhinosinusitis complaints. In The Netherlands, the Lung Alliance Netherlands has made adequate administration of INCs an area of focus since 2019. A standardized protocol for INC administration has been developed on the basis of the existing literature and can be used in daily clinical practice.^10^ Previous research has shown that currently, patients do not know all of the steps for administration of INCs.^11^ This knowledge depends on how instructions are given by the available sources. The instructions about the administration technique that are available in patient information leaflets, via health care providers, and via instruction videos on YouTube are inconsistent and of insufficient quality.^12^, ^13^, ^14^ Complete and uniform instructions regarding the use of INCs are lacking in patient information leaflets.^12^ Most health care workers involved in the care for patients with AR are not able to demonstrate how to administer INCs according the standardized protocol.^13^ The majority of instructional videos on YouTube do not provide patients with instructions according the standardized protocol.^14^ If we want to further improve the quality of administration, it is pivotal to provide patient-tailored instructions, which would help users be better able to learn an adequate administration technique. Therefore, we need to gain insight into the specific characteristics of INC users to identify specific patient groups, which would make it possible to include the needs and wishes of specific patient groups in patient-tailored instructions. Pharmacoepidemiologic research can provide insights into patient characteristics. To the best of our knowledge, there are no recent studies that give insight into characteristics of INC users. The aim of this study was to examine the INC drug prescriptions in The Netherlands in 2019, as obtained from the Dutch population-based prescription InterAction Database (IADB) (available at IADB.nl) of the University of Groningen, to get insight into specific characteristics of INC users.

## Methods

### Data source

For this study, prescription data were obtained from the IADB database of the University of Groningen, The Netherlands. The IADB database is a continuously growing database that contains prescription data from 1996 onward. These prescription data, which are obtained from a number of community pharmacies in The Netherlands, include information about dispensed medication (date of delivery, amount dispensed, daily dose, prescriber, total amount of defined daily doses, and Anatomic Therapeutic Chemical [ATC] class) and information about the individuals to whom the medication is dispensed to (sex and date of birth).^15^ Registration of prescriptions in the database is irrespective of health care insurance and prescriber; however, prescriptions during hospital stays and over-the-counter drugs are not included. In the database, each individual has a unique identifier. The data cannot be linked to an individual. The database covers a population of approximately 1.1 million people. The population in the IADB is representative of the whole Dutch population.^15^

### Study sample and variables

Individuals, independent of age, who received at least 1 INC prescription between January 1, 2015, and December 31, 2019, were included. INCs were defined as ATC class R01AD according to the World Health Organization’s ATC classification system.^16^ Drugs belonging to this class are beclomethasone, budesonide, fluticasone and fluticasone propionate, triamcinolone, and azelastine/fluticasone. In The Netherlands, these drugs are dispensed only on a doctor’s prescription. Moreover, the comedications selected included medications related to treatment of AR, asthma, and atopic dermatitis, namely, systemic antihistamines (class R06), ocular medication (including combinations of cromoglicic acid and antihistamines [class S01GX]), intranasal antihistamines (class R01AC), inhaled antiasthma drugs (class R03) (including inhaled corticosteroids [class R03BA]), and dermal steroids (class D07).

### Data analyses

We performed descriptive analyses to calculate prevalence and incidence rates of INC users in 2019. Prevalence and incidence rates were stratified by age group, with age determined at the dispensing date of the first prescription in 2019. Children were defined as individuals aged 0 to 4, 5 to 9, 10 to 14, and 15 to 18 years; adults were defined as individuals aged 19 to 40 and 41 to 65 years; and elderly individuals were defined as individuals aged 66 years and older. Prevalence and incidence rates were also categorized by sex and subdivided by age group as follows: children (defined as individuals aged 0-10 years), adolescents (defined as individuals aged 11-17 years), and adults (defined as individuals aged ≥18 years). A new (incidental) user was defined as an individual who received an INC prescription after a use-free period of 18 months. Prevalence and incidence rates of INC users were calculated per 1000 individuals, including the 95% CI. To identify continuous and intermittent users, a subanalysis examined the use of INCs in previous years. For this subanalysis, patients with at least 1 INC prescription between January 1, 2019, and December 31, 2019, were included as a starting point. After that, prescription records in the preceding 5 years (prescription written between January 1, 2015, and December 31, 2019) were extracted to determine whether a unique user received a prescription in the preceding year, starting in 2019. Finally, we analyzed comedication of INC users in 2019, including combinations of 2 and 3 drugs.

### Data availability statement

The data sets for this article are not publicly available. Requests to access the data sets should be directed to the first author and will be granted on reasonable request.

### Ethical consent

No ethical consent was needed because the InterAction Database (available at IADB.nl) includes deidentified records and the data have been collected in accordance with the national and European guidelines on privacy requirements and European General Data Protection Regulation (GDPR) for handling human data.

## Results

Demographic data on all individuals included in the IADB prescription database in 2019 are shown in Table I. The total number of INC prescriptions in 2019 was 172,563. These prescriptions were issued to 75,048 individuals, which is equivalent to 2.3 INC prescriptions per individual. The overall prevalence of INC use in 2019 was 68.9 (95% CI = 67.4-70.4) per 1000 individuals. The overall incidence of INC use in 2019 was 25.6 (95% CI = 24.7-26.5) per 1000 individuals.

**Table I:** Baseline demographic data on individuals in 2019

Indicator	Value
Study population, N	1,089,212
Age (y), no. (%)	
0-4	49,451 (4.5)
5-9	51,974 (4.8)
10-14	55,967 (5.1)
15-18	53,176 (4.9)
19-40	293,580 (27.0)
41-65	370,955 (34.1)
≥66	214,109 (19.7)
Sex, no. (%)	
Male, age (y)	
0-10	57,764 (5.3)
11-17	42,193 (3.9)
≥18	433,931 (39.8)
Female, age (y)	
0-10	54,559 (5.0)
11-17	41,499 (3.8)
≥18	459,266 (42.2)

### Prevalence and incidence rates

#### Prevalence

**Fig 1:**
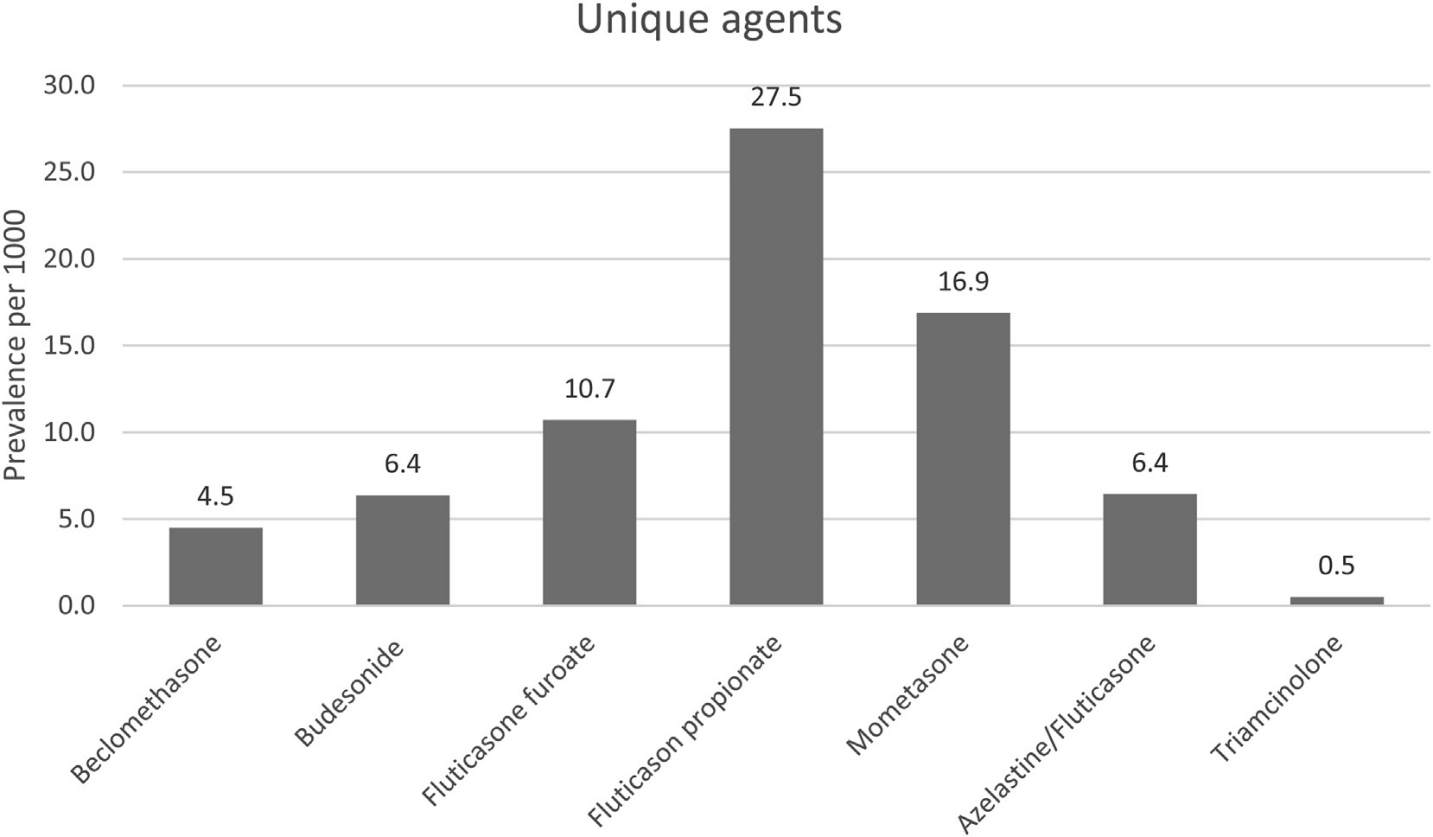
Prevalence rates of the different INC agents in 2019. Prevalence is described per 1000 individuals. Prevalence rates of the unique agents add up to more than the overall prevalence, as 1 person may have had different INC prescriptions.

An overview of the prevalence rates of the different INC agents is given in Fig 1. Fluticasone propionate was prescribed most often (prevalence 27.5 per 1000 individuals). The prevalence rates categorized by age and sex are presented in Table II. Middle-aged adults were more likely to use INCs than those in other age groups were. The prevalence in children was relatively higher in boys than in girls (23.1 per 1000 boys vs 17.0 per 1000 girls). In adolescents, the prevalence appeared to be relatively higher in men than in women (55.5 per 1000 men vs 50.4 per 1000 women). In adults, the prevalence of women was relatively higher than in men (85.2 per 1000 women vs 67.3 per 1000 men).

**Table II:** Prevalence and incidence rates in 2019 (N = 1,089,212)

Indicator	Prevalence				Incidence			
	No. (%)	Per 1000	95% CI		No. (%)	Per 1000	95% CI	
			LL	UL			LL	UL
Overall	75,048	68.9	67.4	70.4	27,856	25.6	24.7	26.5
By age (y)								
0-4	244 (0.3)	4.9	4.3	5.6	133 (0.5)	2.7	2.3	3.2
5-9	1,571 (2.1)	30.2	28.8	31.7	788 (2.8)	15.2	14.1	16.2
10-14	2,643 (3.5)	47.2	45.5	49.0	1,096 (3.9)	19.6	18.5	20.8
15-18	3,056 (4.1)	57.5	55.5	59.5	1,388 (5.0)	26.1	24.8	27.5
19-40	20,812 (27.7)	70.9	70.0	71.8	8,209 (30.5)	28.0	27.4	28.6
41-65	30,921 (41.2)	83.4	82.5	84.2	10,896 (39.1)	29.4	28.8	29.9
≥66	15,801 (21.1)	73.8	72.7	74.9	5,346 (19.2)	25.0	24.3	25.6
By sex and age								
Male, age (y)								
0-10	1,335 (1.8)	23.1	21.9	24.4	634 (2.3)	11.0	10.2	11.9
11-17	2,343 (3.1)	55.5	53.4	57.7	936 (3.4)	22.2	20.8	23.6
≥18	29,201 (38.9)	67.3	66.6	68.0	10,688 (38.4)	24.6	24.2	25.1
Female, age (y)								
0-10	926 (1.2)	17.0	15.9	18.1	468 (1.7)	8.6	7.8	9.4
11-17	2,091 (2.8)	50.4	48.3	52.5	995 (3.6)	24.0	22.5	25.5
≥18	39,152 (52.2)	85.2	84.4	86.1	14,135 (50.7)	30.8	30.3	31.3

*LL,* Lower limit; *UL*, upper limit.

#### Incidence

The incidence rates categorized by age and sex are presented in Table II. Most of the new users were individuals aged 15 to 65 years, followed by elderly individuals aged 66 years and older; and the smallest group of starters was the group consisting of children aged 0 to 14 years. The incidence in children was relatively higher in boys than in girls (11.0 per 1000 boys vs 8.6 per 1000 girls). In adolescents, the incidence appeared to be relatively higher in women than in men (22.5 per 1000 women vs 20.8 per 1000 men). In adults, the prevalence in women was relatively higher than in men (30.8 per 1000 women vs 24.2 per 1000 men).

### Use in previous years

**Fig 2:**
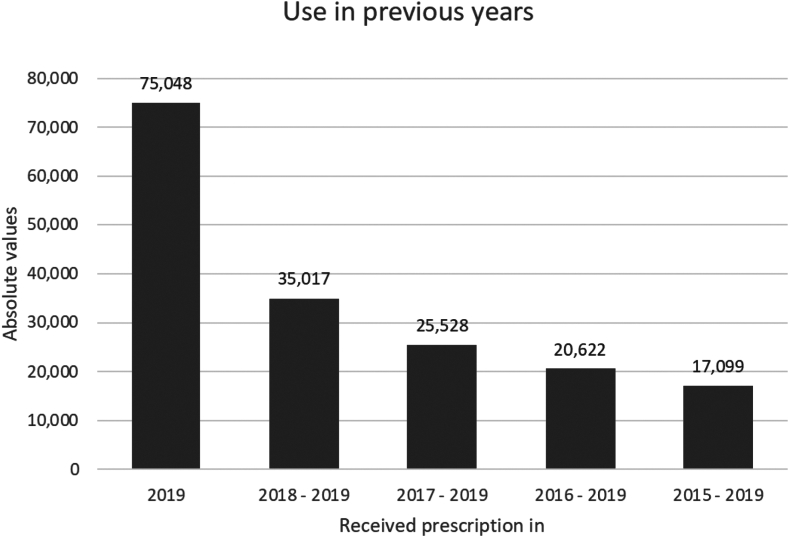
Use of INCs in previous years, with the starting point of users in 2019 to a maximum of 5 years. Results are described as absolute values. Each prevalence rate indicates a prescription in consecutive years: 2019 includes an INC prescription written in 2019; 2018-2019 includes a consecutive INC prescription in 2018 and 2019; 2017-2019 includes a consecutive INC prescription in 2017, 2018, and 2019; 2016-2019 includes a consecutive INC prescription in 2016, 2017, 2018, and 2019; and 2015-2019 includes a consecutive INC prescription in 2015, 2016, 2017, 2018, and 2019. First, the number of users who received an INC prescription in 2019 in the first place was determined in the data set. Next, on the basis of a unique identifier, the number of the users in 2019 who also had a prescription in 2018 was determined. Then, the number of those users who also had a prescription in 2017. This procedure was then repeated for 2016 and 2015. For example, in this figure the those using an INC in 2017-2019 are included in the group of those using an INC in 2018-2019.

The use of INCs in previous years was determined by looking back a maximum of 5 years, with the starting point being users in 2019, as presented in Fig 2. More than half of those using an INC in 2019 did not receive an INC prescription in 2018 (absolute values of 75,048 and 35,017, respectively). Almost a quarter of the INC users in 2019 received an INC prescription in 5 consecutive years (absolute values of 75,048 and 17,099, respectively).

### Comedication

**Fig 3:**
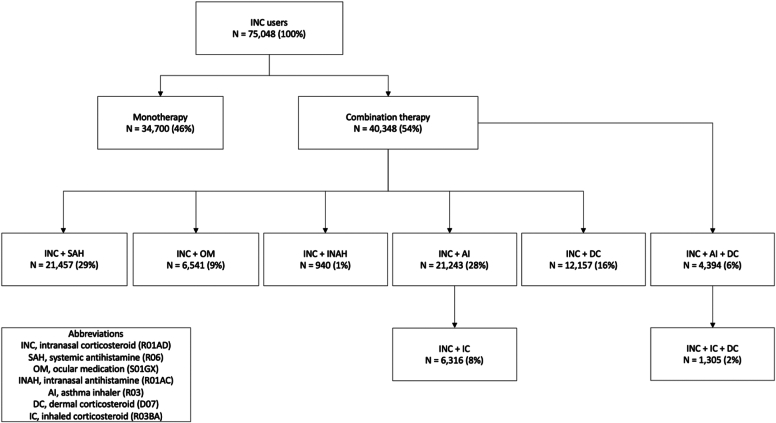
Flowchart of comedication of INC users in 2019.

The majority of INC users (54%) in 2019 used comedication related to AR, asthma, and atopic dermatitis (Fig 3). Approximately half of those using an INC used a systemic antihistamine (29% of all INC users) or an asthma inhaler (28% of all INC users). A dermal corticosteroid was used by 16% of the INC users, an ocular medication was used by 9% of the INC users, and an intranasal antihistamine was used by 1% of the INC users. A combination of an INC, asthma inhaler, and dermal corticosteroid was used by 6% of the INC users. More specifically, a combination of an INC, inhaled corticosteroid, and dermal corticosteroid was used by 2% of the INC users.

## Discussion

### Main findings

The overall prevalence of INC users in 2019 was 68.9 per 1000 individuals, which amounts to 1 in 14 individuals using an INC. The overall incidence was 25.6 per 1000 individuals. The International Study of Asthma and Allergies in Childhood (ISAAC) studies report that in childhood AR prevalence increases with age.^17^ AR complaints tend to decrease in older age groups.^5^ These trends reflect our results despite the fact that INC use cannot be directly related to AR on the basis of the data. We found that the prevalence of INC prescriptions increased with age in childhood from the age of 5 years. The peak prevalence of INC prescriptions was observed in middle-aged individuals (ie, those aged 41-65 years). The prevalence of INC prescriptions decreased with increasing age.

In The Netherlands, INCs are dispensed only on a doctor’s prescription, and the prescription is collected at the pharmacy. Instructions about the administration of INCs to new users may be given by the prescriber during prescribing and by the pharmacist (assistant) during dispensing in the pharmacy during the so-called guidance interview at the first and second dispensing. The incidence rates provided insights into characteristics of new users. Approximately one-third of the total INC users in 2019 (prevalence 68.9 per 1000 individuals) were new INC users (incidence 25.6 per 1000 individuals). It turns out that INCs may be started at any age, with prevalence rates indicating that INC use is distributed among children, adults, and elderly individuals. Attention to age-appropriate instructions would therefore be valuable to being able to provide targeted instructions about the technique for administration of INCs to specific populations. Children may need an approach different from that needed by adults or elderly individuals. Hypothetically, the type of instructions (physical demonstration, written instructions, or visual instructions), as well as the frequency of repetition and evaluation may, be of influence. Considering the fact that each age group includes a relatively large group of users and the proportion of new users is also significant, exploring opportunities for age-appropriate instruction in future research is definitely worthwhile.

Our results indicate that more than half of those using an INC in 2019 did not use an INC in 2018. The reasons for stopping treatment with INCs and whether stopping treatment involved evaluation by a health care worker in consultation with the patient are not always clear. On the one hand, it is possible that the drug was not effective for the patient or the patient experienced side effects.^7^^,^^8^ On the other hand, it is possible that a different diagnosis was made, resulting in a new treatment plan in which the use of INCs has been discontinued. Moreover, owing to the seasonal and fluctuating pattern of AR symptoms, another AR medication or no medication may be sufficient, resulting in an INC prescription not being needed every year. Being able to include the reasons for stopping treatment would be valuable to evaluate treatment adherence, efficacy of treatment, and administration technique. In those cases in which treatment with INCs is stopped owing to insufficient efficacy or side effects, evaluation of the administration technique might provide improvement.

About a quarter of INC users in 2019 collected an INC prescription for at least 5 consecutive years. If an INC is used for a longer period, it is important to determine when the administration technique is evaluated. Longer use may lead to a higher risk of side effects, as a result of which evaluation might be important to prevent potential administration errors that might cause side effects.^18^^,^^19^ International guidelines call for evaluating the inhalation technique of inhalers for asthma and chronic obstructive pulmonary disease at every check-up visit with the doctor.^20^, ^21^, ^22^, ^23^ With regard to INC administration, no advice on this topic is given.^24^

There is an association between AR and allergic asthma, for which the term *united airway disease* is used.^25^ Studies have demonstrated that among patients with asthma and concomitant AR, the risk of subsequent asthma-related events was significantly lower in those who received treatment for AR.^26^ Improvement of INC administration might have beneficial effects in asthma treatment. The prevalence of asthma in patients with AR varies between 10% and 40%.^27^ Our results show that 28% of individuals use an INC in combination with an inhaler. From the data used in this study, it is not possible to conclude that the use of an INC in combination with an inhaler indicates the presence of both conditions, but it does indicate that a relatively large group may benefit from adequate treatment, which might be achieved by proper administration.

Our study demonstrated that 29% of the population of those prescribed an INC use an INC in combination with a systemic antihistamine, 9% use an INC in combination with an ocular medication, and 1% use an INC in combination with an intranasal antihistamine. In cases in which treatment for AR is used, this could indicate that AR symptoms are not fully controlled with INC or systemic antihistamine monotherapy.

Several corticosteroid-containing drugs are used in combination with INCs by 2% to 16% of INC users. Individually, these treatments have been studied extensively and are considered safe, with the risk of side effects being considered low.^28^, ^29^, ^30^, ^31^, ^32^, ^33^, ^34^ However, when these corticosteroid-containing drugs are combined, the risk of more serious, corticosteroid-related side effects (eg, growth retardation, osteoporosis, adrenal cortical hypofunction, and Cushing syndrome) may increase.^18^^,^^19^^,^^35^, ^36^, ^37^, ^38^ Patients, particularly children, are vulnerable for the development of these side effects.^19^^,^^39^ High-quality studies on the cumulative effects of combining these drugs in children are lacking.^18^ In adults, the evidence for a higher risk of side effects is clearer.^19^ Therefore, in daily clinical practice, it is important to monitor these kinds of side effects and, if necessary, share findings with an endocrinologist.

### Implications for health care and future research

Many people are treated with INCs for complaints such as AR or rhinosinusitis with nasal polyps. The fact that patients are insufficiently aware of techniques ensuring adequate INC administration and the fact that administration instructions are of insufficient quality indicate that advances in the area of paying attention to adequate INC administration are lacking in health care and that his situation needs to be improved.^7^, ^8^, ^9^, ^10^, ^11^, ^12^, ^13^ Our results provide insight into the population of patients using INCs and indicate that administration of INCs is being started in patients of all ages. Therefore, it is important to explore opportunities for developing age-appropriate instructions. When receiving instructions, children may need an approach different from that used with adults or elderly individuals. A previous study showed that an age-adjusted instruction video is a useful and easy method to teach children to administer INC sprays correctly.^40^ Therefore, future research exploring the opportunities and efficacy of age-appropriate instructions would be valuable.

It appears that the majority of the population did not receive an INC prescription in 2 consecutive years. Detailed information about the reason for discontinuing treatment is lacking. Future research examining the reasons for stopping INC treatment would be valuable to be able to respond to patients’ needs. In cases in which treatment was stopped because of insufficient efficacy or side effects, evaluation of the administration technique might result in higher efficacy and might prevent side effects. Almost a quarter of the population of patients taking an INC collected an INC prescription for 5 consecutive years and seem to be continuous INC users. Studies have shown that up to 70% of patients with chronic asthma do not use their inhaler correctly. Proper instruction leads to better effect, and instructions must be repeated at least twice.^41^^,^^42^ It is likely that this also applies to INCs. It is important to distinguish between new or intermitted INC users and continuous INC users. Future research needs to clarify whether more attention to when and by whom instructions are given, and focusing this research on the different types of INC users would be beneficial.

Given the fact that an asthma inhaler is used by 28% of INC users, the technique for administration of the asthma inhaler and the INC administration technique should be evaluated both during checkup visits. In addition, because patients receive their prescriptions in the pharmacy, a pharmacist may contribute to better counseling.

A part of the population of those taking an INC used their INC in combination with another corticosteroid-containing drug (an inhaled corticosteroid or dermal corticosteroid). The use of multiple corticosteroid therapies simultaneously remains a point of attention. Corticosteroid-related side effects should be monitored, especially in children.

### Limitations and strengths

When prescription data were used, the indication was missing. Therefore, this study’s results regarding indications should be interpreted with caution. We cannot say with certainty that trends in INCs use correspond with trends in AR prevalence. Also, use of INCs in combination with inhalers is not a necessity for united airway disease. In addition, the use of various antihistamines is likely for treatment of AR; however, this cannot be stated with certainty. However, for most of the findings and implications of this study, it is not necessary to know the indication, as this study focuses on the characteristics of patients using an INC with the aim of providing guidance for improving the administration technique of any INC user, regardless of the indication.

In 2020 and 2021, coronavirus disease 2019 (COVID-19) began to have a major impact on Dutch health care. Given the great uncertainties surrounding COVID-19 control and treatment at the start of the pandemic, we chose to include data on 2019 to avoid having the INC prescriptions for COVID-19 symptoms contaminate the INC prescriptions for other reasons. The data presented might not be completely representative for the current population of those using an INC in 2024.

This is a population-based study in which patients with complaints actually contacted a doctor and that contact resulted in an INC prescription. This provides qualitatively stronger data than data provided by prevalence results from questionnaire-based studies.

### Conclusion

The results of this retrospective study of prescription data obtained from the IADB database give insight into the specific characteristics of INC users, their INC use in previous years, and their comedication. The study gives insights into opportunities for patient-tailored instructions. It appears that INCs are used by children, adults, and elderly individuals. Further research examining the development of age-appropriate instructions would be valuable to determine whether patient-tailored approaches affect treatment adherence and efficacy. Part of this entails zooming in on the distinction between new or intermittent users and continuous users, because more than half of INC users in 2019 did not use an INC in 2018 (new or intermittent users) and about a quarter of INC users in 2019 have collected an INC prescription for at least 5 consecutive years (continuous users). The large proportion of those who stopped taking an INC also indicates the importance of exploring when and by whom the efficacy of INC treatment and the INC administration technique is evaluated. As more than a quarter of the study population used INCs in combination with an inhaler, this study clarifies that evaluation of administration techniques can be combined. In other words, opportunities in which treatment of asthma is combined with evaluation of INC use should be explored. Taken together, these suggestions might lead to a more patient-tailored approach in which individual treatment plans could be developed, which in turn might lead to improved treatment with INCs.

Disclosure of potential conflict of interest: The authors declare that they have no relevant conflicts of interest.

**Key messages**

• Patients’ knowledge about the technique for administration of INCs is insufficient.
• INCs are prescribed for various age groups and types of users, and different types of comedication are used.
• Instruction and evaluation of INC therapy and administration techniques should be more patient tailored.
• Patient-tailored instructions possibly give users the opportunity to better learn an adequate administration technique, resulting in improved treatment.
